# CXCL12 is displayed by rheumatoid endothelial cells through its basic amino-terminal motif on heparan sulfate proteoglycans

**DOI:** 10.1186/ar1900

**Published:** 2006-02-03

**Authors:** Begoña Santiago, Françoise Baleux, Guillermo Palao, Irene Gutiérrez-Cañas, Juan C Ramírez, Fernando Arenzana-Seisdedos, José L Pablos

**Affiliations:** 1 Servicio de Reumatología y Unidad de Investigación, Hospital 12 de Octubre, Avda. de Córdoba s/n, 28041 Madrid, Spain; 2 Organic Chemistry Unit, Pasteur Institute, 28 Rue Dr. Roux, 75724 Paris CEDEX, France; 3 Viral Immunology Unit, Pasteur Institute, 28 Rue Dr. Roux, 75724 Paris CEDEX, France

## Abstract

The chemokine CXCL12 (also known as stromal cell-derived factor, SDF-1) is constitutively expressed by stromal resident cells and is involved in the homeostatic and inflammatory traffic of leukocytes. Binding of CXCL12 to glycosaminoglycans on endothelial cells (ECs) is supposed to be relevant to the regulation of leukocyte diapedesis and neoangiogenesis during inflammatory responses. To improve our understanding of the relevance of this process to rheumatoid arthritis (RA), we have studied the mechanisms of presentation of exogenous CXCL12 by cultured RA ECs. RA synovial tissues had higher levels of CXCL12 on the endothelium than osteoarthritis (OA) tissues; in both, CXCL12 colocalized to heparan sulfate proteoglycans (HSPGs) and high endothelial venules. In cultured RA ECs, exogenous CXCL12α was able to bind in a CXCR4-independent manner to surface HSPGs. Desulfation of RA EC HSPGs by pretreatment with sodium chlorate, or by replacing in a synthetic CXCL12α the residues Lys24 and Lys27 by Ser (CXCL12α-K2427S), decreased or abrogated the ability of the chemokine to bind to RA ECs. *Ex vivo*, synovial ECs from patients with either OA or RA displayed a higher CXCL12-binding capacity than human umbilical vein ECs (HUVECs), and in HUVECs the binding of CXCL12 was increased on exposure to tumor necrosis factor-α or lymphotoxin-α_1_β_2_. Our findings indicate that CXCL12 binds to HSPGs on ECs of RA synovium. The phenomenon relates to the interaction of HSPGs with a CXCL12 domain with net positive surface charge located in the first β strand, which encompasses a canonical BXBB HSPG-binding motif. Furthermore, we show that the attachment of CXCL12 to HSPGs is upregulated by inflammatory cytokines. Both the upregulation of a constitutive chemokine during chronic inflammation and the HSPG-dependent immobilization of CXCL12 in EC surfaces are potential sites for therapeutic intervention.

## Introduction

Chemokines are a large family of soluble proteins involved in leukocyte activation and traffic during inflammatory responses. Chemokines signal through G-protein-coupled receptors [1]. *In vivo*, chemokine-dependent directional migration of leukocytes is supposed to require the immobilization of chemokines either to the extracellular matrix or to cell surfaces. Chemokines induce cell-matrix or cell-cell adhesion through the activation of integrins, and studies *in vivo *demonstrate that the presence of chemokines immobilized on the luminal side of endothelium is a critical step for firm adhesion and transendothelial migration of rolling leukocytes [2-6]. This phenomenon can be reproduced *in vitro *in cultured endothelial cells (ECs) and depends on the addition of exogenous chemokines and the presence of fluid shear-induced mechanical stress on leukocytes [5,6]. Endothelial cells secrete a limited number of chemokines, suggesting that many of the homeostatic or inflammatory chemokines presented at the EC surface come from other cell sources by transcytosis and docking of chemokines on the EC luminal surface [7-9].

CXCL12 (also known as stromal cell-derived factor, SDF-1) is the unique identified natural ligand of the G-protein-coupled receptor CXCR4 and exhibits both homeostatic and proinflammatory functions in humans. The main cellular sources of CXCL12 are resident stromal fibroblasts and epithelial cells. CXCL12 participates in the homeostatic traffic of hematopoietic cells and lymphocytes. Indeed, CXCL12 is constitutively displayed by endothelial cells (ECs) in the bone marrow and secondary lymphoid organs, where it seems to be produced by nearby osteoblasts, stromal cells, or tonsil epithelium [10-12]. The CXCL12/CXCR4 axis also participates in the recruitment of inflammatory cells as shown in animal models of allergic airway disease and rheumatoid arthritis (RA) [13-15]. CXCL12 is constitutively expressed by synovial fibroblasts and lung epithelium and is not a cytokine-inducible factor. The mechanisms that induce the upregulation of CXCL12-mediated leukocyte recruitment in these models therefore remain unclear [15,16]. In this regard, blocking CXCR4 by non-peptidic antagonists has been an effective anti-inflammatory therapy in both asthma and arthritis models [13-15]. In RA and lymph nodes, CXCL12 mRNA is expressed by perivascular stromal cells but not by endothelial cells [11,16]. Our previous studies have shown that CXCL12 protein is specifically immunodetected in RA endothelium, in sharp contrast with normal synovial vessels [16]. The presence of cell-surface immobilized CXCL12 in endotheliawas sensitive to heparitinase, which selectively degrades the glycosaminoglycan moiety (heparan sulfate) in heparan sulfate proteoglycans (HSPGs). These findings suggest that the presence of CXCL12 immobilized in ECs, in the lumen of vessels, is enhanced under inflammatory conditions. Potential mechanisms are either increased secretion or increased transport and docking of perivascular CXCL12 to the luminal side of ECs. Previous reports showed that, *in vitro*, CXCL12 specifically binds heparan sulfates through a domain with net positive surface charge located in its first β strand, which encompasses a canonical BXBB HSPG-binding motif [17-19]. The sensitivity of EC-bound CXCL12 to heparitinase in RA is compatible with cell-surface attachment of the chemokine by a HSPG-dependent mechanism.

We have analyzed the mechanisms of interaction between exogenous CXCL12 and ECs obtained from synovial tissues, and here we show that CXCL12 binds to membrane HSPGs in cultured RA ECs independently of its CXCR4 receptor. Importantly, the amount of immobilized CXCL12 was upregulated by tumor necrosis factor (TNF)-α or lymphotoxin α_1_β_2 _(LT-α_1_β_2_) treatment in non-synovial ECs, suggesting that the presentation of CXCL12 on ECs can be upregulated at the EC level, independently of its degree of expression. Furthermore, the immobilization of CXCL12 on RA EC surfaces is explained by the selective interaction of sulphated HSPGs and no other proteoglycans. This interaction relies on the integrity of a positively charged amino acid cluster located in the first sheet of the CXCL12 β strand, which forms the canonical binding motif for HSPG.

## Materials and methods

Biotinylated CXCL12α was synthesized by the Merrifield solid-phase method on a fully automated peptide synthesizer using Fmoc (*N*-(9-fluorenyl)methoxycarbonyl) chemistry as described previously [17,18]. Selective biotinylation at the carboxy-terminal position was achieved by incorporating a lysine residue (Lys68) bearing a 4,4-dimethyl-2,6-dioxocyclohex-1-ethylidene (Dde) protective group on the side chain. Coupling of biotin was performed on the peptide resin after Dde deprotection. Non-biotinylated native CXCL12α or 2/6 CXCL12α, in which the basic residues Lys24 and Lys27 were replaced by Ser (CXCL12α-K2427S), were synthesized similarly.

Synovial EC cultures were obtained from enzymatic cell suspensions of three RA and four osteoarthritis (OA) synovial tissues obtained at the time of joint replacement surgery. All patients gave informed consent, and the study was approved by the ethics committee of the Hospital 12 de Octubre. ECs were purified by two rounds of immunomagnetic enrichment with anti-CD105 coupled to magnetic beads (Miltenyi Biotech, Bergisch Gladbach, Germany). ECs were cultured in medium199 (Life Technologies, Paisley, Renfrewshire, Scotland) with 10% FCS and the endothelial identity of cultured cells was confirmed by flow cytometry with UEA-rhodamine and anti-P1H12 antibodies (Chemicon, Temecula, CA, USA). Human umbilical vein ECs (HUVECs) were prepared from umbilical cord by digestion with collagenase and were propagated in medium199 with 20% FCS. Cultures displaying more than 90% cells positive for both EC markers were used between the third and seventh passages.

Cultured ECs were exposed to 300 to 1,000 nM CXCL12α peptides for 90 minutes in PBS buffer at 4°C and washed extensively in PBS. Surface presentation of exogenous CXCL12α was analysed by flow cytometry with K15C mAb and fluorescein isothiocyanate (FITC)-labelled secondary antibody or, in the case of biotinylated CXCL12α, with avidin-FITC (Pharmingen, San Diego, CA, USA). Expression of HSPGs in cultured ECs was studied with 10E4 anti-heparan sulfate-FITC mAb (Calbiochem, San Diego, CA, USA). Expression of CXCR4 in cultured ECs was studied with 12G5 mAb (Pharmingen) in 0.5% Tween 20 permeabilized or non-permeabilized ECs.

Where indicated, 500 μg/ml sodium heparin (Rovi S.A., Madrid, Spain) or 50 μg/ml T134 (a CXCR4 inhibitor) was added to the CXCL12α incubation medium. T134 specifically prevents the binding of CXCL12 to CXCR4 receptor at picomolar concentrations [20]. Pretreatment of EC cultures with 60 mM sodium chlorate was performed for 24 hours before CXCL12α binding. Removal of surface HSPGs in cultured cells was performed by treatment with a cocktail of 100 mU/ml heparitinases I, II and III each, or 100 mU/ml chondroitin sulfate ABC lyase as a control for 90 minutes at 37°C (Sigma Aldrich Química S.A., Madrid, Spain). Where indicated, EC cultures were stimulated with either 25 ng/ml TNF-α or 10 ng/ml LT-α1/β2 (R&D Systems, Inc., Abingdon, Oxon, UK) for 16 hours before CXCL12α binding.

CXCL12 expression by ECs was studied by RT-PCR on cDNA synthesized from 1 μg of total RNA with the use of the *CXCL12 *oligonucleotides 5' -TCTGAGAGCTCGCTTGAGTG-3' (upstream) and 5' -GTGGATCGCATCTATGCATG-3' (downstream) and the β-actin oligonucleotides 5' -CTACCTCATGAAGATCCTCAC-3' (upstream) and 5' -GTCCACGTCACACTTCATGATG-3' (downstream). As a positive control for *CXCL12 *expression we used cDNA from RA synovial fibroblasts.

For immunolabelling of synovial tissues from patients with RA and control patients with OA, tissues were snap-frozen in optimal cutting temperature (OCT) compound and stored at -80°C. Double labelling for HSPGs and CXCL12, or MECA-79 and CXCL12, was performed as described previously [16,21].

Flow cytometry data were expressed as mean fluorescence intensity (mean ± SD), normalized by the mean fluorescence intensity of the negative control. Statistical analysis was performed with Student's *t *test.

## Results

### HSPGs and CXCL12 are immunodetected in RA vessels

**Figure 1:**
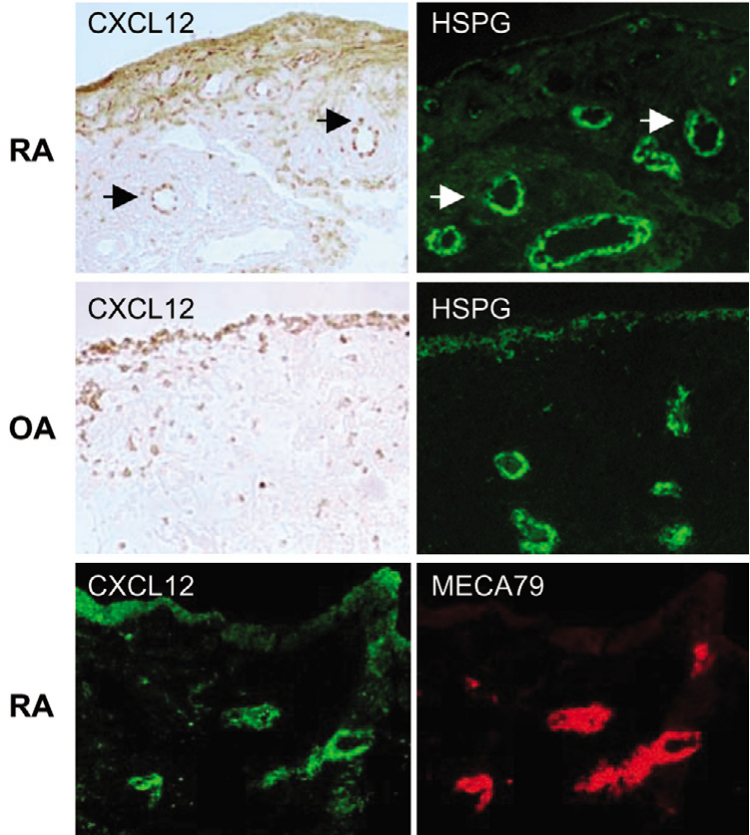
Double labelling of CXCL12/HSPGs and CXCL12/MECA-79 of rheumatoid arthritis (RA) and osteoarthritis (OA) synovial tissues. Frozen RA or OA sections were labeled simultaneously with anti-CXCL12 K15C mAb and fluorescein isothiocyanate (FITC)- conjugated anti-heparan sulfate proteoglycan (HSPG) mAb (green fluorescence) or MECA-79 mAb (red fluorescence). In CXCL12/HSPG double-labeled sections, CXCL12 was developed with immunoperoxidase (brown color) and in CXCL12/MECA-79 double-labeled sections with a secondary FITC-labeled antibody. Arrows indicate colocalization of CXCL12 to HSPG-labeled RA vessels. The same section, sequentially photographed under appropriate optics, is shown in parallel left and right panels. Original magnification × 400.

By immunoperoxidase labelling of RA (*n *= 9) and OA (*n *= 8) synovial sections, we found that CXCL12 was abundantly present on the luminal side of RA sublining vessels (nine of nine tissues) in contrast to OA vessels, in which it was only rarely (three of eight tissues) and weakly detected (Figure 1). HSPGs were detected with a diffuse pattern involving all vessels in both RA and OA sections. Colocalization of HSPGs and CXCL12 was confirmed in RA vessels, although many vessels in RA sections and all vessels in most OA sections lacked CXCL12 despite their clear expression of HSPGs (Figure 1). In both RA and OA sections, CXCL12 was detected in lining synoviocytes and scattered fibroblasts in the sublining, although RA sections showed a higher proportion of labelled cells and some areas of extracellular matrix labelling.

High endothelial venule (HEV)-like vessels are found in RA synovium but not in OA synovium and represent specialized vessels involved in cell recruitment [21]. Because labeling of RA vessels with CXCL12 seemed to be present in both flat and HEVs, we studied whether MECA-79-expressing HEVs also display CXCL12 immunostaining. By double MECA-79 and CXCL12 immunofluorescent labelling, we observed that MECA-79-positive HEVs preferentially displayed CXCL12 compared with flat MECA-79-negative vessels (Figure 1).

### CXCL12α binding to cultured RA ECs

**Figure 2:**
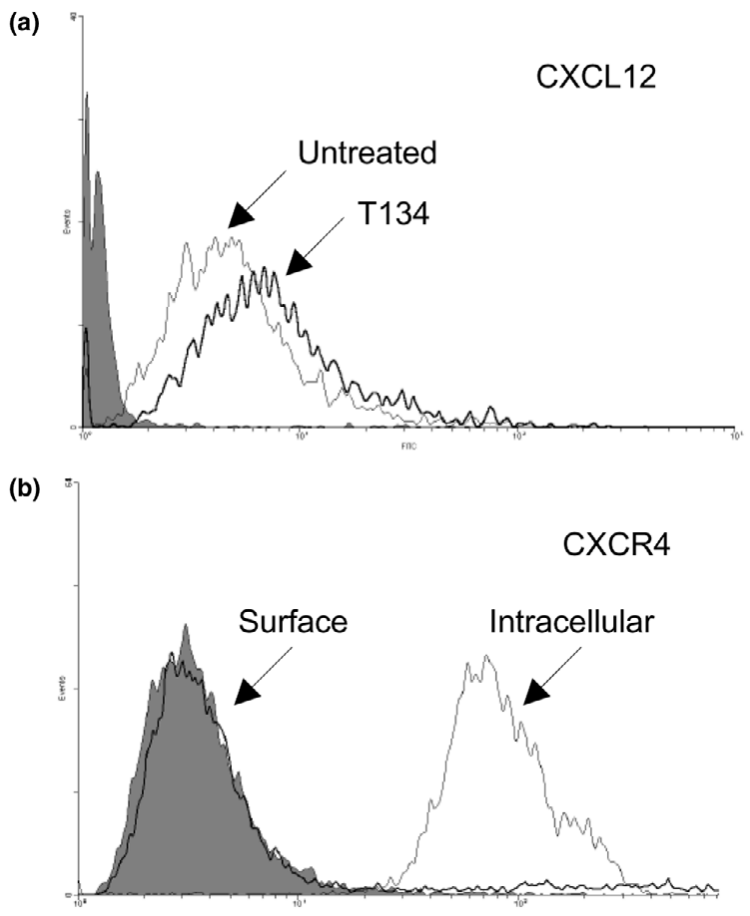
Binding of CXCL12 to RA ECs is independent of CXCR4. **(a) **Rheumatoid arthritis endothelial cells (RA ECs) were incubated with 300 nM biotinylated CXCL12α and, after extensive washing to remove free chemokine, were labeled with fluorescein isothiocyanate-conjugated avidin. Where indicated, RA ECs were simultaneously incubated with 50 μg/ml of the CXCR4 antagonist T134 or not (untreated). **(b) **Surface or intracellular CXCR4 was detected with 12G5 mAb. Filled histograms show isotype control IgG. Results are representative of three independent experiments with RA ECs from different donors.

To study the potential interactions between CXCL12α and EC surface molecules, we analysed the ability of human RA ECs to present exogenous native or biotinylated CXCL12α on their surface molecules. Endogenous CXCL12 expression was not detectable by the K15C mAb in cultured RA ECs. We have previously shown by *in situ *hybridization that, in spite of the positive immunodetection of CXCL12 in RA ECs, these cells lacked CXCL12 mRNA expression [16]. To confirm the lack of CXCL12 expression by RA ECs we performed RT-PCR in cultured RA ECs, which yielded negative results in all tested RA EC cell lines; this was in contrast with synovial fibroblasts, in which CXCL12 message was detected (data not shown). We found that after the addition of exogenous native or biotinylated CXCL12α, surface-bound CXCL12α was readily detected by flow cytometry (Figure 2a). This binding was unlikely to have been due to CXCR4 binding, as indicated by the following lines of evidence. In cultured RA ECs, CXCR4 was largely located to an intracellular pool and surface expression was very low or undetectable by flow cytometric analysis with 12G5 anti-CXCR4 mAb of permeabilized or non-permeabilized cells (Figure 2b). Moreover, CXCR4-bound CXCL12 cannot be detected by K15C, which recognizes an epitope encoding the critical residues involved in CXCL12 cell signalling [17]. Finally, pretreatment of RA ECs with an excess of the CXCR4-specific inhibitor T134, which precludes CXCL12α binding to CXCR4 [20], did not affect the immunodetection of RA EC-bound CXCL12 (Figure 2a).

**Figure 3:**
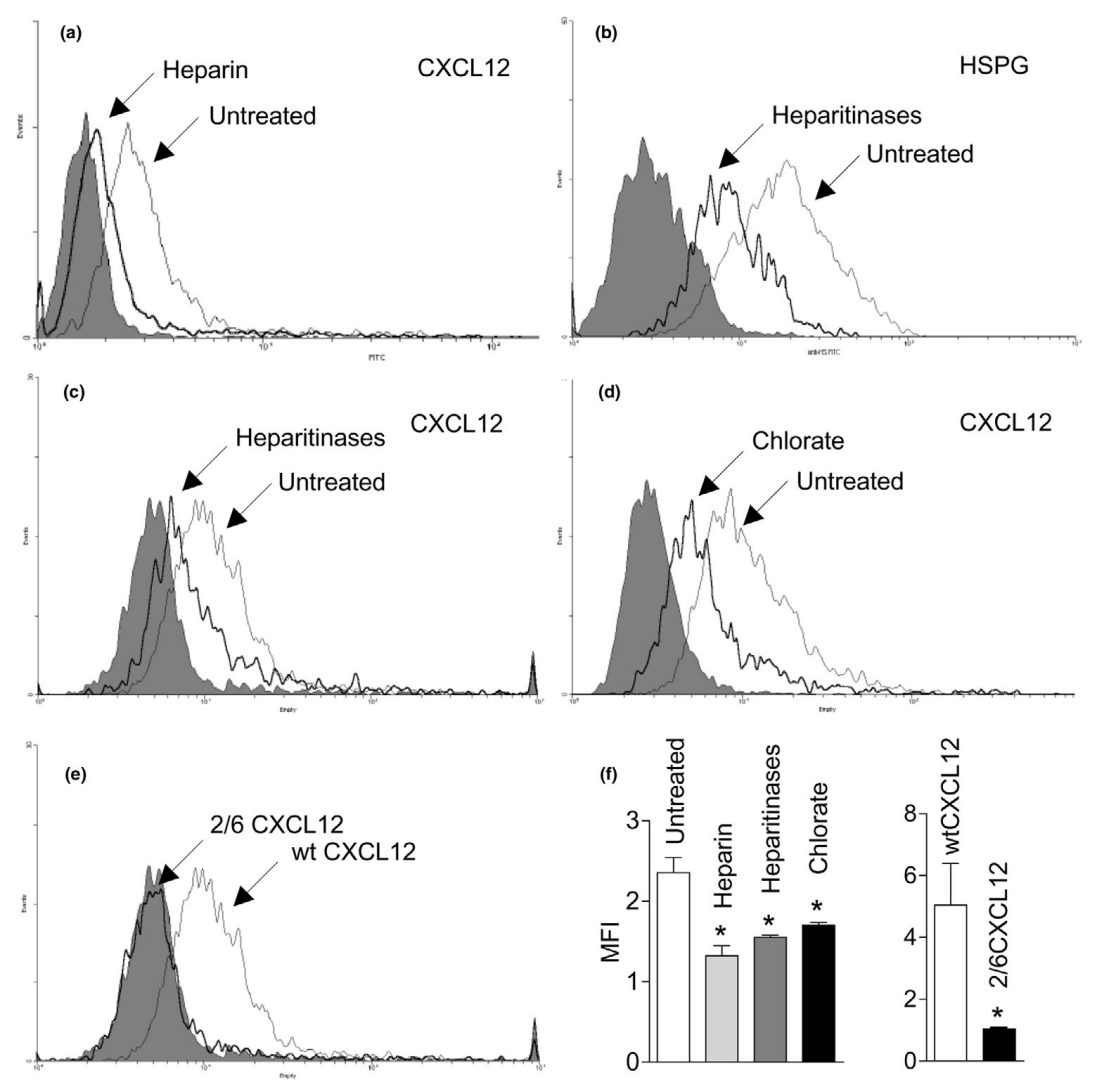
Binding of CXCL12 peptides to HSPG on RA ECs. Rheumatoid arthritis endothelial cells (RA ECs) were incubated with 300 nM biotinylated CXCL12α and, after extensive washing to remove free chemokine, were labeled with fluorescein isothiocyanate-conjugated avidin. Where indicated, RA ECs were simultaneously incubated with soluble sodium heparin **(a) **or pretreated with heparitinases **(b, c) **or sodium chlorate **(d)**. In (b), surface heparan sulfate proteoglycan (HSPG) was detected with 10E4 mAb in RA ECs pretreated or not with heparitinases. **(e) **RA ECs were incubated with 1 μM non-biotinylated wild-type CXCL12α or 2/6 CXCL12α and labelled with K15C mAb. Filled histograms show isotype control IgG. Results are representative of three to five independent experiments with RA ECs from three different donors. **(f) **Summary of normalized mean fluorescence intensity (MFI) data. Error bars show SD. *p < 0.05.

Heparin is a sulfated GAG, chemically related to HSPGs, that has previously been shown to interact with CXCL12α *in vitro *[18]. Because soluble heparin decreased the binding of CXCL12α to RA ECs (Figure 3a), we pretreated RA ECs either with heparitinases I, II, and III or with chondroitin sulfate lyase to assess whether RA EC HSPGs were involved in the interaction. Pretreatment with heparitinases, but not chondroitinase, substantially decreased the level of HSPG expression and surface binding of CXCL12α to RA ECs (Figure 3b,c).

To examine whether the interaction between RA EC HSPGs and CXCL12α requires the cluster of basic residues that comprise a putative HSPG-binding motif in the first amino-terminal β strand of CXCL12 and sulfated groups on HSPGs as previously observed in filter binding assays [18], we compared the binding of wild-type CXCL12α and 2/6 CXCL12α (K2427S mutation) to RA ECs. Detection of surface-bound wild-type CXCL12α or 2/6 CXCL12α was performed with anti-CXCL12α K15C antibody, which recognizes both peptides equally [17]. Modified 2/6 CXCL12α was undetectable on RA ECs, in contrast with wild-type CXCL12α (Figure 3e). To analyse the role of HSPG sulfate groups, we preincubated RA ECs for 24 hours with sodium chlorate, a specific inhibitor of the synthesis of 3' -phosphoadenosine 5' -phosphosulfate (PAPS), which is the sole sulfur donor in sulphated HSPG synthesis. Pretreatment with sodium chlorate decreased the binding of CXCL12α to RA ECs, confirming the need for sulphate groups in the interaction between CXCL12α and HSPG (Figure 3d).

### CXCL12α binding to different synovial and non-synovial ECs and cytokine effects

The observed interaction between EC HSPGs and CXCL12α is not specific to RA ECs, because it has been reported in other human ECs such as primary or transformed HUVECs [17,19]. The increased CXCL12 endothelial labelling observed in RA tissues by immunohistoshemistry might therefore relate to a higher amount of exogenous CXCL12 docking onto RA vessels, but it might also relate to specific changes in ECs present in the inflammatory environment, as suggested by our observation of stronger CXCL12 display by HEV-type vessels.

**Figure 4:**
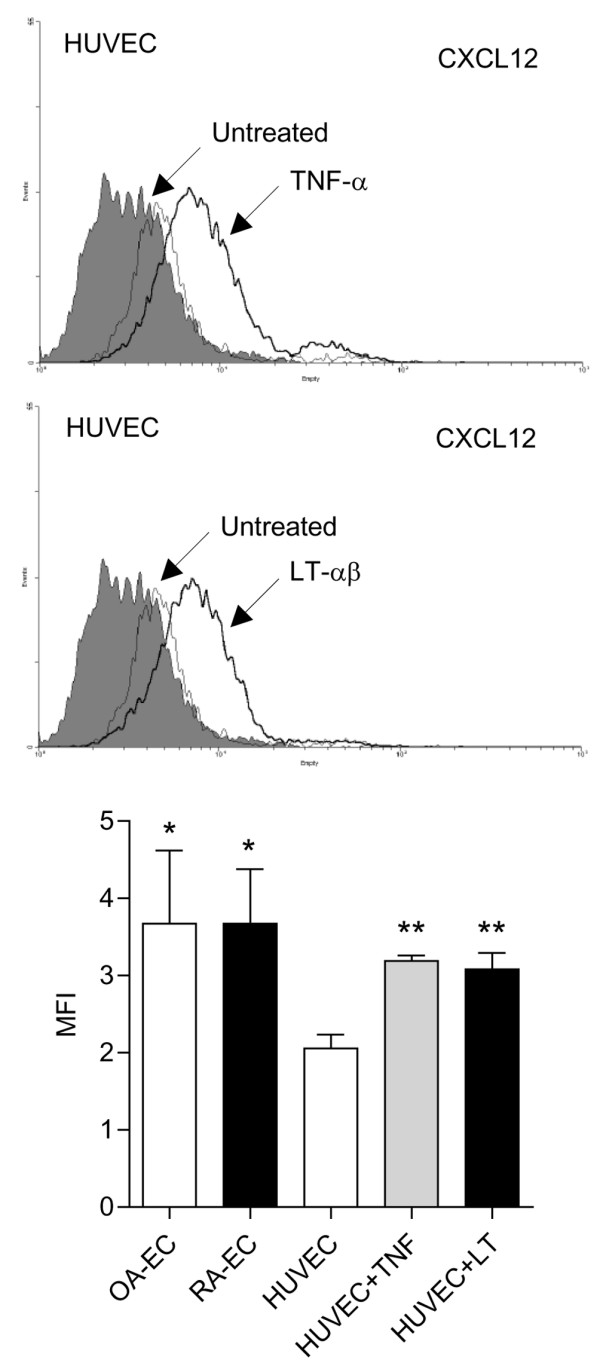
Binding of CXCL12 to synovial osteoarthritis (OA) and rheumatoid arthritis (RA) ECs and cytokine-treated HUVECs. Endothelial cells (ECs) were incubated with 300 nM biotinylated CXCL12α and, after extensive washing to remove free chemokine, were labeled with fluorescein isothiocyanate-conjugated avidin. Human umbilical-vein endothelial cells (HUVECs) were treated with tumor necrosis factor-α (TNF-α; top) or lymphotoxin α_1_β_2 _(LT-αβ; middle) for 16 hours before CXCL12α binding as indicated. Filled histograms show isotype control IgG. The bottom panel shows a summary of normalized mean fluorescence intensity (MFI) data. Error bars show SD. Results are representative of three to five independent experiments in three RA EC, four OA EC and three HUVEC lines. *p < 0.05 compared with HUVECs, **p < 0.05 compared with cytokine-untreated HUVECs.

The capacity of cultured RA ECs to bind biotinylated CXCL12α was similar to that of OA ECs but in contrast to that of non-synovial ECs (HUVECs), in which constitutive binding of biotinylated CXCL12α was significantly lower than observed in synovial RA or OA ECs (Figure 4). To test whether CXCL12α binding can be upregulated in cells with lower binding capacity, we pretreated HUVECs with either TNF-α or LT-α_1_β_2_, two cytokines involved in the activation of ECs during chronic inflammation. Pretreatment of HUVECs with TNF-α or LT-α_1_β_2 _significantly increased the capacity of HUVECs to bind exogenous CXCL12α to HUVECs (Figure 4). Constitutive or TNF-α-induced binding of CXCL12α to HUVECs was also dependent on HSPGs because it was not competed for by T134 and was decreased by pretreatment with sodium chlorate, heparin, or heparitinases (data not shown).

Finally, the possibility of the involvement of MECA-79-related glycoproteins in CXCL12α binding *in vitro *was excluded, because under culture conditions neither RA ECs nor HUVECs expressed MECA-79 (data not shown).

## Discussion

Several observations have indicated the importance of chemokine docking on the luminal surface of ECs on the chemokine activation of firm adhesion and transendothelial migration of circulating leukocytes [2-6]. The lack of expression of several homeostatic and inflammatory chemokines by ECs contrasts with their relevance in the homing of leukocytes and other cell types to central lymphoid organs or inflamed tissues, suggesting that the presentation of exogenous chemokines by ECs is a pathophysiologically important process [7-11].

CXCL12 has been suggested to have multifunctional roles in the pathogenesis of RA, and its genetic variation is associated with a more severe disease [16,22,23]. Two of the proposed effects of CXCL12 in the pathogenesis of RA are cell recruitment and angiogenesis, two processes requiring its presence on ECs, which do not express autocrine CXCL12 [16,22]. Our data demonstrate that CXCL12α, which is produced only by RA stromal cells *in vivo*, is specifically bound to HSPGs on RA ECs, from where it may trigger the firm adhesion and transendothelial migration of rolling leukocytes, or may contribute to angiogenesis by enhanced interactions with EC CXCR4. Consistently with previous studies in a human EC cell line [17], CXCL12α docking on RA EC membrane relies on interactions between its first β strand basic motif and sulphated HSPG groups. This process was not specific to RA ECs because it is observed in other synovial (OA) and non-synovial (HUVEC) EC types. Interestingly, a higher CXCL12 binding capacity was observed *ex vivo *in all lines of synovial origin (either OA or RA) compared with non-synovial ECs (HUVECs), and this may relate to features specific to either the tissue or vessel type.

Importantly, the preferential location of CXCL12 in rheumatoid HEVs decorated with the MECA-79 epitope, as a marker for L-selectin ligand, provides a link between selectin and chemokine-mediated adhesion processes. Both phenomena may be a consequence of the exposure of EC to the cytokines TNF-α and LT-α_1_β_2_, which, in transgenic animals or *in vitro*, have been shown to induce the expression of synthetic enzymes for the sulfation of HSPGs and MECA-79 as well as homing chemokines [21,24-26]. In the present study, CXCL12 binding was increased *in vitro *in HUVECs exposed to TNF-α or LT-α_1_β_2_, suggesting that inducible changes in the affinity or composition of HSPGs might account for this phenomenon in ECs from a non-inflammatory environment in response to these cytokines. Previous studies show that HSPG sulfation, and the enzymes responsible for it, are inducible by cytokines [24,25]. These data and our previous observations suggest that the elements needed for L-selectin rolling [21], and for CXCL12 presentation (which can induce firm adhesion and transendothelial migration of rolling cells during homeostatic traffic to lymphoid organs), are coupled downstream of the TNF-α or LT-α_1_β_2 _cytokines and are reproduced in chronically inflamed tissues.

Because HSPG expression is a widespread feature of normal or pathological synovial vessels, the need for integrity of the basic motif on the N-terminal end of CXCL12 as well as the sulfation of HSPGs provides potential targets for intervention. In this regard, sulphated heparin or soluble sulphated oligosacharide derivatives may interfere specifically with the presentation of CXCL12 on ECs [27]. This approach has been experimentally addressed in another CXCL12-related process, the homing of hematopoietic precursors to the bone marrow [10]. In this model, the treatment of animals with fucoidan or related sulphated oligosacharides increases circulating CXCL12α and reduces its function in the bone marrow, resulting in the increased mobilization of hematopoietic cells [28,29]. In different inflammatory settings, limited information suggests that heparin or derivatives can also have therapeutic effects [30], although studies on CXCL12 function in this context are yet to be performed. The administration of mutant chemokines unable to bind to HSPGs has also been proposed as an alternative therapeutic approach [31,32].

## Conclusion

Our observations demonstrate interactions between CXCL12 and HSPGs in human RA ECs and suggest that this process is enhanced in the endothelium of a chronically inflammatory environment and *in vitro *under the influence of cytokines, providing a potential point of intervention to downregulate CXCL12 functions on circulating leukocytes.

## Abbreviations

EC = endothelial cell; FCS = fetal calf serum; FITC = fluorescein isothiocyanate; HEV = high endothelial venule; HSPG = heparan sulfate proteoglycan; HUVEC = human umbilical vein EC; LT-α_1_β_2 _= lymphotoxin α_1_β_2_; mAb = monoclonal antibody; OA = osteoarthritis; RA = rheumatoid arthritis; RT-PCR = reverse transcriptase polymerase chain reaction; TNF-α = tumor necrosis factor-α.

## Competing interests

The authors declare that they have no competing interests.

## Authors' contributions

BS performed flow cytometry and immunofluorescence studies, FB and FAS prepared the peptides and antibodies used and contributed to drafting and reviewing the manuscript, GP and IGC contributed to the collection and culture of human EC lines, JCR participated in the design of the study and data analysis, and JLP conceived of the study and participated in its design and coordination. All authors read and approved the final manuscript.
